# Separation of Membrane Vesicles and Cytosol from Cultured Cells and Bacteria in a Preformed Discontinuous Gradient

**DOI:** 10.1100/tsw.2002.834

**Published:** 2002-06-07

**Authors:** John Graham

**Affiliations:** School of Biomolecular Sciences, Liverpool John Moores University, UK

**Keywords:** protein localization, cytosol, membrane vesicles, cultured cells, bacteria, OptiPrep^TM^, iodixanol, discontinuous gradient, flotation

## Abstract

There are many situations when it is necessary to separate rapidly and efficiently a cytosolic and a membrane vesicle fraction from either cultured cells or from bacteria. Flotation of the vesicles through a low-density barrier from a dense sample zone using the low viscosity medium iodixanol allows complete separation of these compartments. As the sample is exposed to the gmax the tendency of the proteins to sediment overcomes any diffusion in the opposite direction.

**METHOD TYPE:** extraction, isolation, purification and separation

**SUB METHOD TYPE:** centrifugation

## INTRODUCTION

There are many situations where it is necessary to provide an efficient separation of membrane vesicles from cytosolic proteins. Both permeabilization of cultured cells to release cytoplasmic vesicles of the exocytic pathway[1] and the isolation of vesicles budded from the plasma membrane by selective hypoosmotic cell disruption[2] require subsequent resolution of these vesicles and the cytosol which is also released by the treatments.

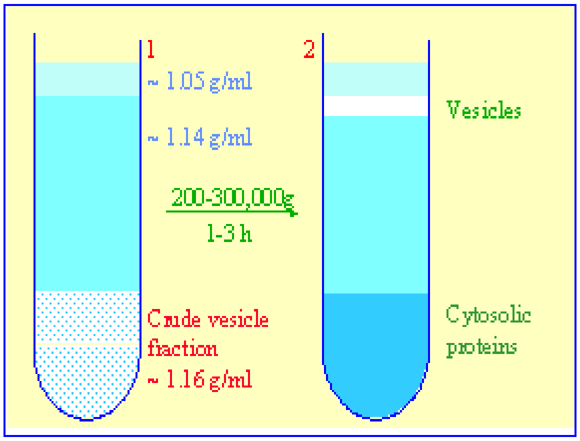

**FIGURE 1.** Separation of vesicles and cytosolic proteins from mammalian cultured cells by flotation in a discontinuous iodixanol gradient.

A number of protocols have been published which all rely on the same strategy; i.e., flotation of the vesicles through a discontinuous gradient of iodixanol from a dense load zone. Since proteins are considerably more dense (approx. 1.26 g/ml) in iodixanol than are membrane vesicles from cultured mammalian cells (generally <1.13 g/ml), if the crude vesicle-containing fraction is adjusted to 30% iodixanol (approx. 1.16 g/ml) and overlayered with 25% iodixanol (approx. 1.14 g/ml) then the vesicles will float through the lower density layer and the proteins will tend to sediment from the load zone. This is an ideal way of separating the two compartments, which are separated by the low-density layer.

Although sucrose gradients might be used in the same mode of operation, (a) they are more viscous and consequently particles move more slowly and (b) as the density of the vesicles is much higher (because of the high osmolality of the sucrose solutions) the difference in density between the vesicles and the proteins is considerably less.

These problems are particularly severe with bacterial cytoplasmic membrane vesicles. Their density is higher because of the higher protein/lipid ratio in such membranes compared to those from mammalian cells. De Leeuw et al.[3] used a similar OptiPrep™ flotation strategy for the purification of inverted cytoplasmic vesicles to study the localization of FtsY in *Escherichia coli*, but increased the iodixanol concentration in two layers to 44 and 30%.

As with all flotation methods, a small volume of buffer or 5–10% iodixanol is always layered on top of the low-density barrier to prevent banding of the vesicles at an air/liquid interface. The strategy is described in Fig. 1.

An alternative strategy using a self-generated iodixanol gradient has been devised for yeast cells[4] and this can be adapted to other cell types. As a continuous gradient is generated above the bottom-loaded sample, it may provide some additional fractionation of the vesicle population.

## MATERIALS AND EQUIPMENT

OptiPrep™ (60% w/v, iodixanol)

OptiPrep™ Diluent: this is used to prepare a Working Solution (WS) containing 30 or 50% (w/v) iodixanol together with the appropriate concentrations of buffer and other additives (see Notes 1 and 2)

WS Diluent: this is mixed with the WS to produce iodixanol gradient solutions containing appropriate concentrations of buffer and other additives (see Note 1). It is normally the medium used to suspend the crude vesicles

Include protease inhibitors in the diluents as required

Microcentrifuge or low-speed centrifuge with swinging-bucket rotor (5–10 ml tubes)
Ultracentrifuge with swinging-bucket rotor (e.g., Beckman SW55 or Sorvall TH660). The procedure can be scaled down or up as a required. For smaller volumes use the Beckman TLS55; for larger volumes use the Beckman SW41 or Sorvall TH641.
Syringe and metal cannula (for underlayering) and/or plastic Pasteur pipette (for overlayering)

## METHOD

Carry out all operations at 0–4°C.

### Cultured Cells

This protocol is adapted from Refs. [1,2].

1. Remove cells from the vesicle-containing suspension either in a microcentrifuge or by centrifugation at 1000*g* for 5 min in a standard low-speed centrifuge.
2. Aspirate the supernatant and adjust it to 30% (w/v) iodixanol by thorough mixing with the 50% (w/v) iodixanol WS (see Notes 1 and 2).
3. Prepare solutions containing 25 and 5% (w/v) iodixanol by diluting the 50% iodixanol WS with WS Diluent (see Note 1).
4. In tubes for a swinging-bucket rotor, layer 2 ml each of the crude vesicle fraction in 30% iodixanol and the 25% iodixanol and fill the tube by overlayering with 5% iodixanol.
5. Centrifuge at approx 250,000*g* for 3 h.
6. Using a syringe collect the vesicles that band at the top interface (see Fig. 1) and, if required, the bottom layer containing cytosolic proteins. Alternatively the gradient can be collected in a series of fractions by tube puncture, upward displacement with a dense medium or aspiration from the bottom[5].

### Bacteria

This protocol is adapted from Ref. [3].

1. Adjust the crude vesicle preparation in WS Diluent to 44% iodixanol by addition of a 50% iodixanol WS (see Note 3).
2. Transfer to tubes for the chosen swinging-bucket rotor and overlayer with approx. 5 vol of 30% iodixanol (see Note 3).
3. Fill the tubes by overlayering with WS Diluent and centrifuge at approx 170,000*g* for 3 h.
4. Using a syringe, collect the vesicles that band at the top interface (see Fig. 1) and, if required, the bottom layer containing cytosolic proteins. Alternatively the gradient can be collected in a series of fractions by tube puncture, upward displacement with a dense medium or aspiration from the bottom[5].

## ANALYSIS

Spectrophotometric (above 340 nm) analysis of enzymes, SDS-PAGE, and immunoprecipitation can be carried out in the presence of iodixanol. None of the common enzyme markers for membranes are inhibited by iodixanol[6]. If, however, because the vesicles or proteins are not at a sufficiently high concentration for analysis, or if some particular functional inhibition is apparent, then the iodixanol can be easily removed. Vesicles suspensions should be diluted with 2 vol of HB, to reduce the density and viscosity of the suspension and after sedimentation at 100,000–150,000*g* for 45 min, the pellet can be suspended in an appropriately small volume of buffer. Removal of iodixanol from soluble proteins is best achieved by ultrafiltration through microcentrifuge cones, such as those in the Vectaspin® range manufactured by Whatman.

## NOTES

1. For the isolation of budded vesicles[2], all of the iodixanol solutions contain 10 mM Hepes-NaOH (pH 7.4), 140 mM KCl, 2 mM EGTA, 1 mM DTT. This is the WS Diluent. The easiest way of achieving this is to dilute 5 vol of OptiPrep™ with 1 vol of OptiPrep™ Diluent containing 6× the concentration of these reagents to produce a 50% iodixanol WS. The WS is then further diluted with WS Diluent or mixed with the crude vesicle fraction to raise its density. The same strategy can be applied to the formation of any gradient solutions. For the isolation of vesicles from permeabilized cells[1] the vesicles are suspended in 30% (w/v) iodixanol containing 60 mM KCl, 10 mM Hepes-NaOH (pH 7.2), 2.5 mM MgOAc. Love et al.[1] used 25% and 10% (w/v) iodixanol for the two lower density layers, both of which contain 150 mM KCl, 10 mM Hepes-NaOH (pH 7.2), 2.5 mM MgOAc. See Refs. [7,8] for more information on preparing gradient solutions.
2. Rather than adjusting the crude vesicle fraction to 30% iodixanol by the addition of a 50% iodixanol WS, Love et al.[1] first pelleted the vesicles and then suspended them directly in 30% iodixanol. Either strategy should be effective.
3. The WS Diluent contains 50 mM Hepes-NaOH (pH 7.6), 500 mM KOAc, 5 mM MgOAc. The WS contains 50% (w/v) iodixanol, 0.25 M sucrose, 50 mM Hepes-NaOH (pH 7.6), 500 mM KOAc, 5 mM MgOAc. Dilute WS with WS Diluent to prepare a 30% iodixanol solution. Suspend crude bacterial cytoplasmic membrane vesicles in 15 µl of WS Diluent and mix with 105 µl of WS. Overlay with 580 µl of the 30% iodixanol solution and 300 µl of WS Diluent. Carry out the separation in a Beckman TLS55 rotor or scale up as required[3].

## ACKNOWLEDGEMENTS

The author and TheScientificWorld wish to thank Axis-Shield PoC, AS, Oslo, Norway for their kind permission to adapt OptiPrep™ Application Sheet S28 in the preparation of this Protocol Article.
